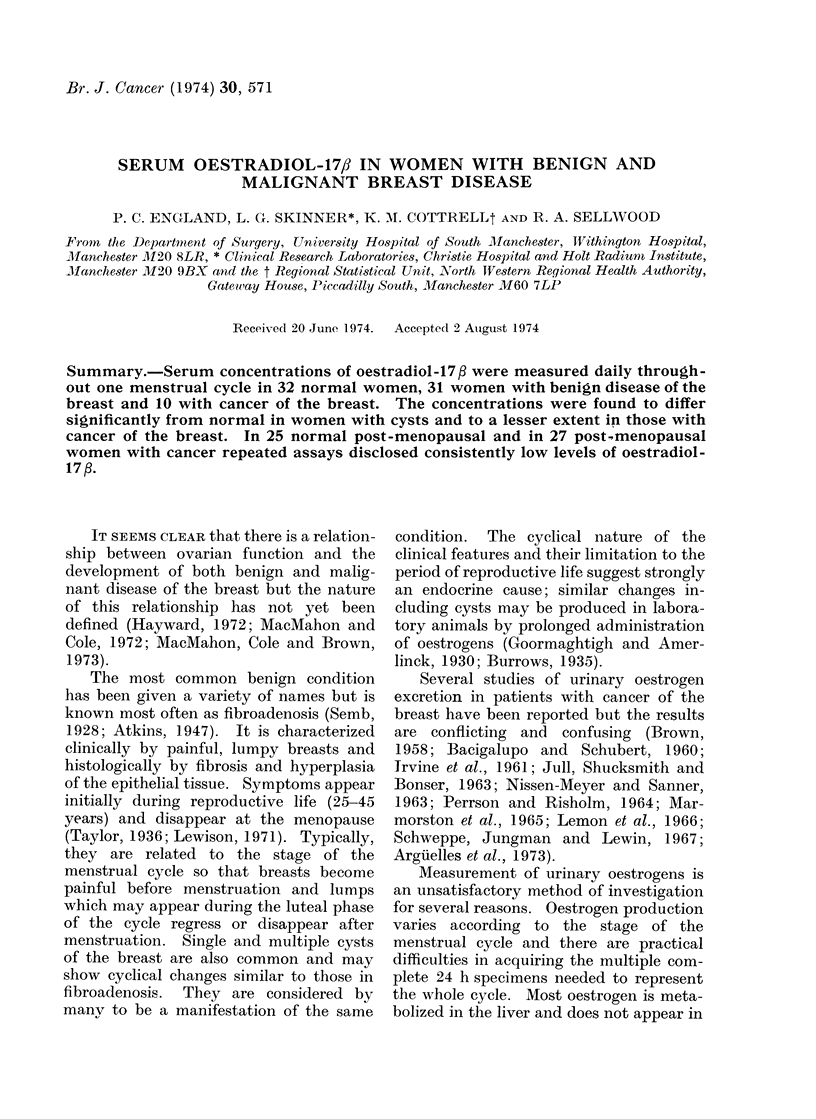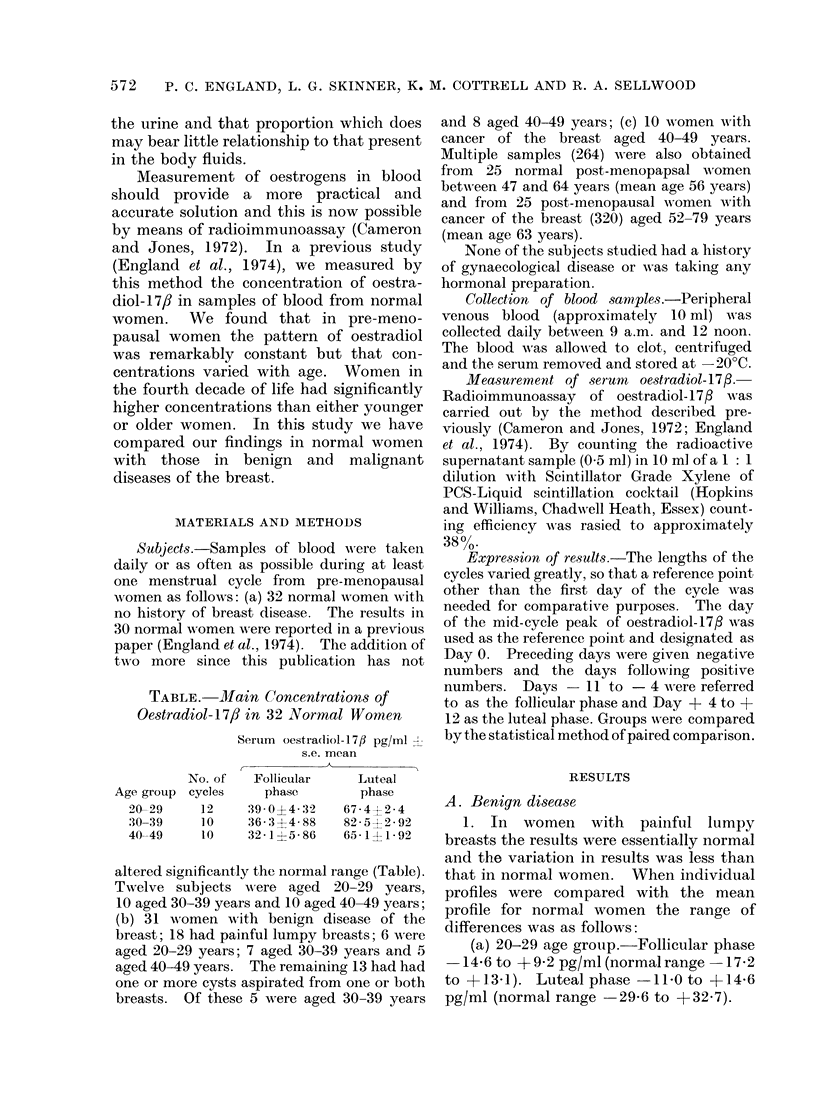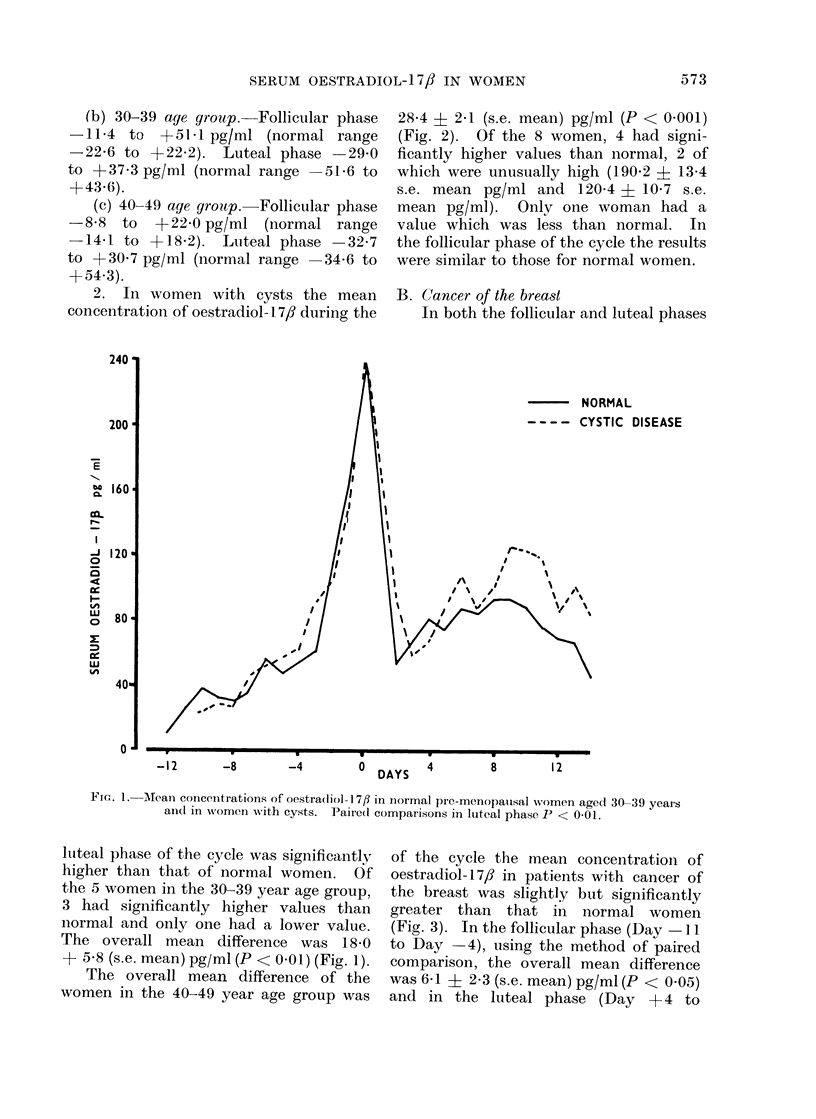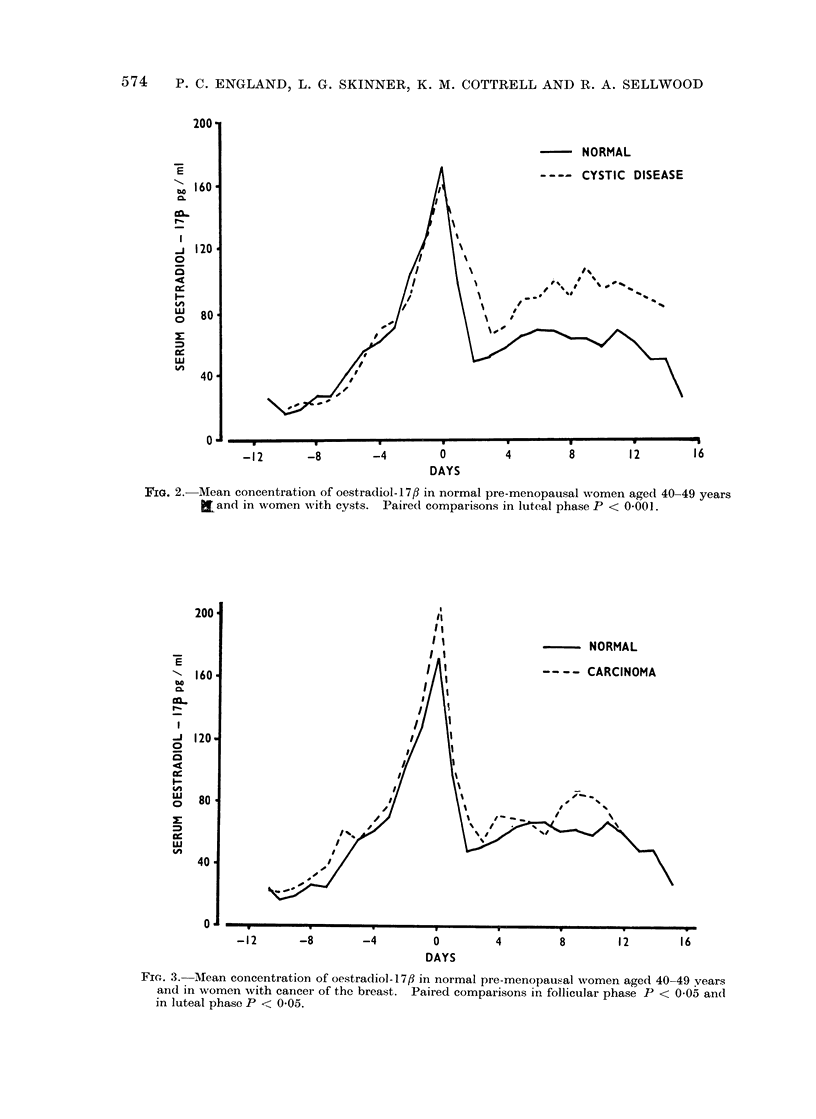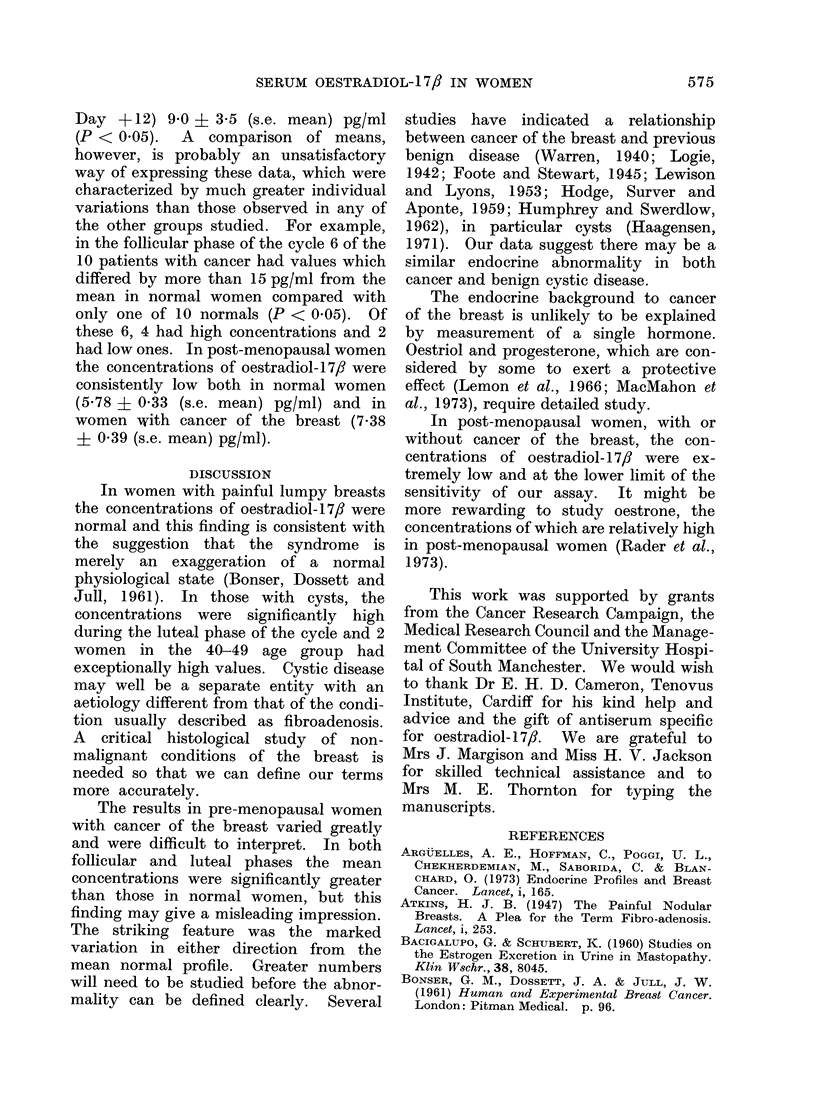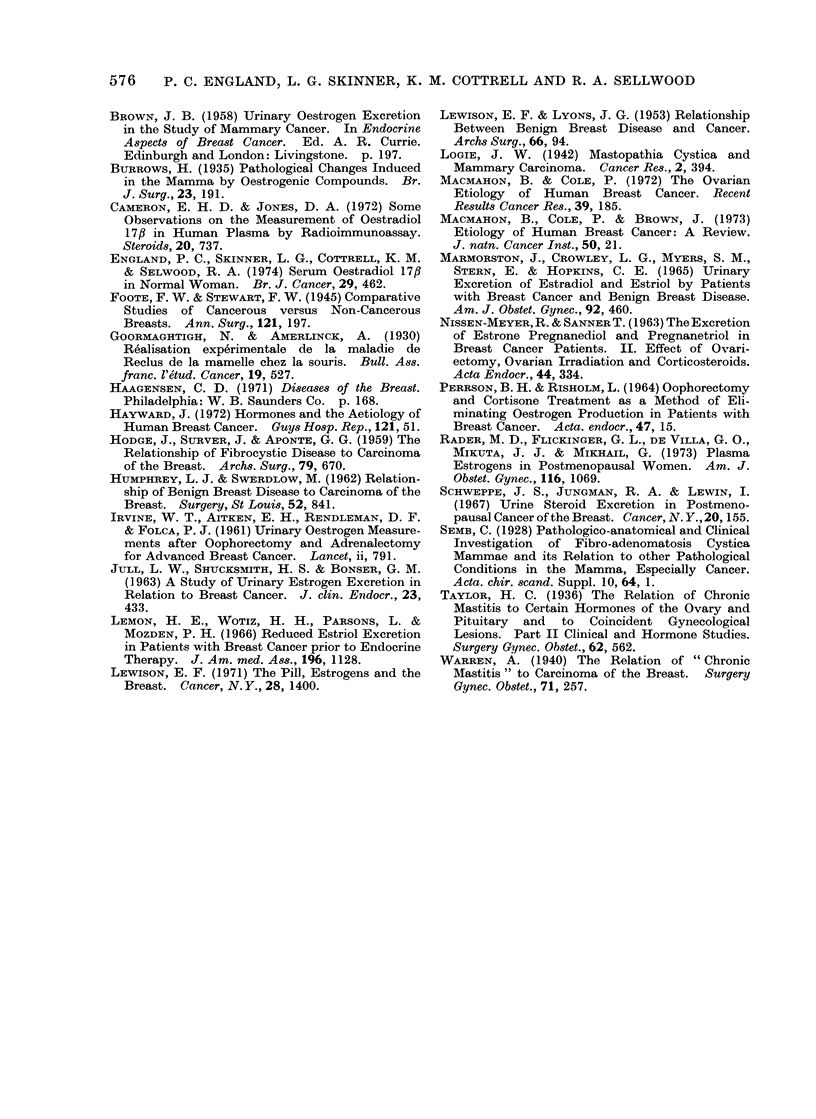# Serum Oestradiol-17β in Women with Benign and Malignant Breast Disease

**DOI:** 10.1038/bjc.1974.237

**Published:** 1974-12

**Authors:** P. C. England, L. G. Skinner, K. M. Cottrell, R. A. Sellwood

## Abstract

Serum concentrations of oestradiol-17β were measured daily throughout one menstrual cycle in 32 normal women, 31 women with benign disease of the breast and 10 with cancer of the breast. The concentrations were found to differ significantly from normal in women with cysts and to a lesser extent in those with cancer of the breast. In 25 normal post-menopausal and in 27 post-menopausal women with cancer repeated assays disclosed consistently low levels of oestradiol-17β.


*Br. J. Cancer* (1974) **30**, 571

### SERUM OESTRADIOL-17β IN WOMEN WITH BENIGN AND MALIGNANT BREAST DISEASE

P. C. ENGLAND, L. G. SKINNER*, K. M. COTTRELL† AND R. A. SELLWOOD

*From the Department of Surgery, University Hospital of South Manchester, Withington Hospital, Manchester M20 8LR, * Clinical Research Laboratories, Christie Hospital and Holt Radium Institute, Manchester M20 9BX and the † Regional Statistical Unit, North Western Regional Health Authority, Gateway House, Piccadilly South, Manchester M60 7LP*

Received 20 June 1974. Accepted 2 August 1974

**Summary.—Serum concentrations of oestradiol-17β were measured daily throughout one menstrual cycle in 32 normal women, 31 women with benign disease of the breast and 10 with cancer of the breast. The concentrations were found to differ significantly from normal in women with cysts and to a lesser extent in those with cancer of the breast. In 25 normal post-menopausal and in 27 post-menopausal women with cancer repeated assays disclosed consistently low levels of oestradiol-17β.**

It seems clear that there is a relationship between ovarian function and the development of both benign and malignant disease of the breast but the nature of this relationship has not yet been defined (Hayward, 1972; MacMahon and Cole, 1972; MacMahon, Cole and Brown, 1973).

The most common benign condition has been given a variety of names but is known most often as fibroadenosis (Semb, 1928; Atkins, 1947). It is characterized clinically by painful, lumpy breasts and histologically by fibrosis and hyperplasia of the epithelial tissue. Symptoms appear initially during reproductive life (25–45 years) and disappear at the menopause (Taylor, 1936; Lewison, 1971). Typically, they are related to the stage of the menstrual cycle so that breasts become painful before menstruation and lumps which may appear during the luteal phase of the cycle regress or disappear after menstruation. Single and multiple cysts of the breast are also common and may show cyclical changes similar to those in fibroadenosis. They are considered by many to be a manifestation of the same condition. The cyclical nature of the clinical features and their limitation to the period of reproductive life suggest strongly an endocrine cause; similar changes including cysts may be produced in laboratory animals by prolonged administration of oestrogens (Goormaghtigh and Amerlinck, 1930; Burrows, 1935).

Several studies of urinary oestrogen excretion in patients with cancer of the breast have been reported but the results are conflicting and confusing (Brown, 1958; Bacigalupo and Schubert, 1960; Irvine *et al.*, 1961; Jull, Shucksmith and Bonser, 1963; Nissen-Meyer and Sanner, 1963; Perrson and Risholm, 1964; Marmorston *et al.*, 1965; Lemon *et al.*, 1966; Schweppe, Jungman and Lewin, 1967; Argüelles *et al.*, 1973).

Measurement of urinary oestrogens is an unsatisfactory method of investigation for several reasons. Oestrogen production varies according to the stage of the menstrual cycle and there are practical difficulties in acquiring the multiple complete 24 h specimens needed to represent the whole cycle. Most oestrogen is metabolized in the liver and does not appear in the urine and that proportion which does may bear little relationship to that present in the body fluids.

Measurement of oestrogens in blood should provide a more practical and accurate solution and this is now possible by means of radioimmunoassay (Cameron and Jones, 1972). In a previous study (England *et al.*, 1974), we measured by this method the concentration of oestradiol-17β in samples of blood from normal women. We found that in pre-menopausal women the pattern of oestradiol was remarkably constant but that concentrations varied with age. Women in the fourth decade of life had significantly higher concentrations than either younger or older women. In this study we have compared our findings in normal women with those in benign and malignant diseases of the breast.

### MATERIALS AND METHODS

*Subjects.*—Samples of blood were taken daily or as often as possible during at least one menstrual cycle from pre-menopausal women as follows: (a) 32 normal women with no history of breast disease. The results in 30 normal women were reported in a previous paper (England *et al.*, 1974). The addition of two more since this publication has not altered significantly the normal range (Table). Twelve subjects were aged 20–29 years, 10 aged 30–39 years and 10 aged 40–49 years; (b) 31 women with benign disease of the breast; 18 had painful lumpy breasts; 6 were aged 20–29 years; 7 aged 30–39 years and 5 aged 40–49 years. The remaining 13 had had one or more cysts aspirated from one or both breasts. Of these 5 were aged 30–39 years and 8 aged 40–49 years; (c) 10 women with cancer of the breast aged 40–49 years. Multiple samples (264) were also obtained from 25 normal post-menopapsal women between 47 and 64 years (mean age 56 years) and from 25 post-menopausal women with cancer of the breast (320) aged 52–79 years (mean age 63 years).

TABLE.—*Main Concentrations of Oestradiol*-17β *in* 32 *Normal Women*

| | | Serum oestradiol-17β pg/ml ± s.e. mean | |
|---|---|---|---|
| Age group | No. of cycles | Follicular phase | Luteal phase |
| 20–29 | 12 | 39·0±4·32 | 67·4±2·4 |
| 30–39 | 10 | 36·3±4·88 | 82·5±2·92 |
| 40–49 | 10 | 32·1±5·86 | 65·1±1·92 |

None of the subjects studied had a history of gynaecological disease or was taking any hormonal preparation.

*Collection of blood samples.*—Peripheral venous blood (approximately 10 ml) was collected daily between 9 a.m. and 12 noon. The blood was allowed to clot, centrifuged and the serum removed and stored at −20°C.

*Measurement of serum oestradiol*-17β.—Radioimmunoassay of oestradiol-17β was carried out by the method described previously (Cameron and Jones, 1972; England *et al.*, 1974). By counting the radioactive supernatant sample (0·5 ml) in 10 ml of a 1 : 1 dilution with Scintillator Grade Xylene of PCS-Liquid scintillation cocktail (Hopkins and Williams, Chadwell Heath, Essex) counting efficiency was rasied to approximately 38%.

*Expression of results.*—The lengths of the cycles varied greatly, so that a reference point other than the first day of the cycle was needed for comparative purposes. The day of the mid-cycle peak of oestradiol-17β was used as the reference point and designated as Day 0. Preceding days were given negative numbers and the days following positive numbers. Days − 11 to − 4 were referred to as the follicular phase and Day + 4 to + 12 as the luteal phase. Groups were compared by the statistical method of paired comparison.

### RESULTS

### *A. Benign disease*

1. In women with painful lumpy breasts the results were essentially normal and the variation in results was less than that in normal women. When individual profiles were compared with the mean profile for normal women the range of differences was as follows:

(a) 20–29 age group.—Follicular phase −14·6 to +9·2 pg/ml (normal range −17·2 to +13·1). Luteal phase −11·0 to +14·6 pg/ml (normal range −29·6 to +32·7).

(b) 30–39 *age group*.—Follicular phase −11·4 to +51·1 pg/ml (normal range −22·6 to +22·2). Luteal phase −29·0 to +37·3 pg/ml (normal range −51·6 to +43·6).

(c) 40–49 *age group*.—Follicular phase −8·8 to +22·0 pg/ml (normal range −14·1 to +18·2). Luteal phase −32·7 to +30·7 pg/ml (normal range −34·6 to +54·3).

2. In women with cysts the mean concentration of oestradiol-17β during the luteal phase of the cycle was significantly higher than that of normal women. Of the 5 women in the 30–39 year age group, 3 had significantly higher values than normal and only one had a lower value. The overall mean difference was 18·0 + 5·8 (s.e. mean) pg/ml ($P < 0·01$) (Fig. 1).

The overall mean difference of the women in the 40–49 year age group was 28·4 ± 2·1 (s.e. mean) pg/ml ($P < 0·001$) (Fig. 2). Of the 8 women, 4 had significantly higher values than normal, 2 of which were unusually high (190·2 ± 13·4 s.e. mean pg/ml and 120·4 ± 10·7 s.e. mean pg/ml). Only one woman had a value which was less than normal. In the follicular phase of the cycle the results were similar to those for normal women.

B. *Cancer of the breast*

In both the follicular and luteal phases of the cycle the mean concentration of oestradiol-17β in patients with cancer of the breast was slightly but significantly greater than that in normal women (Fig. 3). In the follicular phase (Day −11 to Day −4), using the method of paired comparison, the overall mean difference was 6·1 ± 2·3 (s.e. mean) pg/ml ($P < 0·05$) and in the luteal phase (Day +4 to

FIG. 1.—Mean concentrations of oestradiol-17β in normal pre-menopausal women aged 30–39 years and in women with cysts. Paired comparisons in luteal phase $P < 0·01$.

FIG. 2.—Mean concentration of oestradiol-17β in normal pre-menopausal women aged 40–49 years and in women with cysts. Paired comparisons in luteal phase $P < 0{\cdot}001$.

FIG. 3.—Mean concentration of oestradiol-17β in normal pre-menopausal women aged 40–49 years and in women with cancer of the breast. Paired comparisons in follicular phase $P < 0{\cdot}05$ and in luteal phase $P < 0{\cdot}05$.

Day +12) $9 \cdot 0 \pm 3 \cdot 5$ (s.e. mean) pg/ml ($P < 0 \cdot 05$). A comparison of means, however, is probably an unsatisfactory way of expressing these data, which were characterized by much greater individual variations than those observed in any of the other groups studied. For example, in the follicular phase of the cycle 6 of the 10 patients with cancer had values which differed by more than 15 pg/ml from the mean in normal women compared with only one of 10 normals ($P < 0 \cdot 05$). Of these 6, 4 had high concentrations and 2 had low ones. In post-menopausal women the concentrations of oestradiol-17$\beta$ were consistently low both in normal women ($5 \cdot 78 \pm 0 \cdot 33$ (s.e. mean) pg/ml) and in women with cancer of the breast ($7 \cdot 38 \pm 0 \cdot 39$ (s.e. mean) pg/ml).

### DISCUSSION

In women with painful lumpy breasts the concentrations of oestradiol-17$\beta$ were normal and this finding is consistent with the suggestion that the syndrome is merely an exaggeration of a normal physiological state (Bonser, Dossett and Jull, 1961). In those with cysts, the concentrations were significantly high during the luteal phase of the cycle and 2 women in the 40–49 age group had exceptionally high values. Cystic disease may well be a separate entity with an aetiology different from that of the condition usually described as fibroadenosis. A critical histological study of non-malignant conditions of the breast is needed so that we can define our terms more accurately.

The results in pre-menopausal women with cancer of the breast varied greatly and were difficult to interpret. In both follicular and luteal phases the mean concentrations were significantly greater than those in normal women, but this finding may give a misleading impression. The striking feature was the marked variation in either direction from the mean normal profile. Greater numbers will need to be studied before the abnormality can be defined clearly. Several studies have indicated a relationship between cancer of the breast and previous benign disease (Warren, 1940; Logie, 1942; Foote and Stewart, 1945; Lewison and Lyons, 1953; Hodge, Surver and Aponte, 1959; Humphrey and Swerdlow, 1962), in particular cysts (Haagensen, 1971). Our data suggest there may be a similar endocrine abnormality in both cancer and benign cystic disease.

The endocrine background to cancer of the breast is unlikely to be explained by measurement of a single hormone. Oestriol and progesterone, which are considered by some to exert a protective effect (Lemon *et al.*, 1966; MacMahon *et al.*, 1973), require detailed study.

In post-menopausal women, with or without cancer of the breast, the concentrations of oestradiol-17$\beta$ were extremely low and at the lower limit of the sensitivity of our assay. It might be more rewarding to study oestrone, the concentrations of which are relatively high in post-menopausal women (Rader *et al.*, 1973).

This work was supported by grants from the Cancer Research Campaign, the Medical Research Council and the Management Committee of the University Hospital of South Manchester. We would wish to thank Dr E. H. D. Cameron, Tenovus Institute, Cardiff for his kind help and advice and the gift of antiserum specific for oestradiol-17$\beta$. We are grateful to Mrs J. Margison and Miss H. V. Jackson for skilled technical assistance and to Mrs M. E. Thornton for typing the manuscripts.

### REFERENCES

Argüelles, A. E., Hoffman, C., Poggi, U. L., Chekherdemian, M., Saborida, C. & Blanchard, O. (1973) Endocrine Profiles and Breast Cancer. *Lancet*, i, 165.

Atkins, H. J. B. (1947) The Painful Nodular Breasts. A Plea for the Term Fibro-adenosis. *Lancet*, i, 253.

Bacigalupo, G. & Schubert, K. (1960) Studies on the Estrogen Excretion in Urine in Mastopathy. *Klin Wschr.*, **38**, 8045.

Bonser, G. M., Dossett, J. A. & Jull, J. W. (1961) *Human and Experimental Breast Cancer.* London: Pitman Medical. p. 96.

BROWN, J. B. (1958) Urinary Oestrogen Excretion in the Study of Mammary Cancer. In *Endocrine Aspects of Breast Cancer*. Ed. A. R. Currie. Edinburgh and London: Livingstone. p. 197.

BURROWS, H. (1935) Pathological Changes Induced in the Mamma by Oestrogenic Compounds. *Br. J. Surg.*, **23**, 191.

CAMERON, E. H. D. & JONES, D. A. (1972) Some Observations on the Measurement of Oestradiol 17$\beta$ in Human Plasma by Radioimmunoassay. *Steroids*, **20**, 737.

ENGLAND, P. C., SKINNER, L. G., COTTRELL, K. M. & SELWOOD, R. A. (1974) Serum Oestradiol 17$\beta$ in Normal Woman. *Br. J. Cancer*, **29**, 462.

FOOTE, F. W. & STEWART, F. W. (1945) Comparative Studies of Cancerous versus Non-Cancerous Breasts. *Ann. Surg.*, **121**, 197.

GOORMAGHTIGH, N. & AMERLINCK, A. (1930) Réalisation expérimentale de la maladie de Reclus de la mamelle chez la souris. *Bull. Ass. franc. l'étud. Cancer*, **19**, 527.

HAAGENSEN, C. D. (1971) *Diseases of the Breast*. Philadelphia: W. B. Saunders Co. p. 168.

HAYWARD, J. (1972) Hormones and the Aetiology of Human Breast Cancer. *Guys Hosp. Rep.*, **121**, 51.

HODGE, J., SURVER, J. & APONTE, G. G. (1959) The Relationship of Fibrocystic Disease to Carcinoma of the Breast. *Archs. Surg.*, **79**, 670.

HUMPHREY, L. J. & SWERDLOW, M. (1962) Relationship of Benign Breast Disease to Carcinoma of the Breast. *Surgery, St Louis*, **52**, 841.

IRVINE, W. T., AITKEN, E. H., RENDLEMAN, D. F. & FOLCA, P. J. (1961) Urinary Oestrogen Measurements after Oophorectomy and Adrenalectomy for Advanced Breast Cancer. *Lancet*, ii, 791.

JULL, L. W., SHUCKSMITH, H. S. & BONSER, G. M. (1963) A Study of Urinary Estrogen Excretion in Relation to Breast Cancer. *J. clin. Endocr.*, **23**, 433.

LEMON, H. E., WOTIZ, H. H., PARSONS, L. & MOZDEN, P. H. (1966) Reduced Estriol Excretion in Patients with Breast Cancer prior to Endocrine Therapy. *J. Am. med. Ass.*, **196**, 1128.

LEWISON, E. F. (1971) The Pill, Estrogens and the Breast. *Cancer, N.Y.*, **28**, 1400.

LEWISON, E. F. & LYONS, J. G. (1953) Relationship Between Benign Breast Disease and Cancer. *Archs Surg.*, **66**, 94.

LOGIE, J. W. (1942) Mastopathia Cystica and Mammary Carcinoma. *Cancer Res.*, **2**, 394.

MACMAHON, B. & COLE, P. (1972) The Ovarian Etiology of Human Breast Cancer. *Recent Results Cancer Res.*, **39**, 185.

MACMAHON, B., COLE, P. & BROWN, J. (1973) Etiology of Human Breast Cancer: A Review. *J. natn. Cancer Inst.*, **50**, 21.

MARMORSTON, J., CROWLEY, L. G., MYERS, S. M., STERN, E. & HOPKINS, C. E. (1965) Urinary Excretion of Estradiol and Estriol by Patients with Breast Cancer and Benign Breast Disease. *Am. J. Obstet. Gynec.*, **92**, 460.

NISSEN-MEYER, R. & SANNER T. (1963) The Excretion of Estrone Pregnanediol and Pregnanetriol in Breast Cancer Patients. II. Effect of Ovariectomy, Ovarian Irradiation and Corticosteroids. *Acta Endocr.*, **44**, 334.

PERRSON, B. H. & RISHOLM, L. (1964) Oophorectomy and Cortisone Treatment as a Method of Eliminating Oestrogen Production in Patients with Breast Cancer. *Acta. endocr.*, **47**, 15.

RADER, M. D., FLICKINGER, G. L., DE VILLA, G. O., MIKUTA, J. J. & MIKHAIL, G. (1973) Plasma Estrogens in Postmenopausal Women. *Am. J. Obstet. Gynec.*, **116**, 1069.

SCHWEPPE, J. S., JUNGMAN, R. A. & LEWIN, I. (1967) Urine Steroid Excretion in Postmenopausal Cancer of the Breast. *Cancer, N.Y.*, **20**, 155.

SEMB, C. (1928) Pathologico-anatomical and Clinical Investigation of Fibro-adenomatosis Cystica Mammae and its Relation to other Pathological Conditions in the Mamma, Especially Cancer. *Acta. chir. scand.* Suppl. 10, **64**, 1.

TAYLOR, H. C. (1936) The Relation of Chronic Mastitis to Certain Hormones of the Ovary and Pituitary and to Coincident Gynecological Lesions. Part II Clinical and Hormone Studies. *Surgery Gynec. Obstet.*, **62**, 562.

WARREN, A. (1940) The Relation of "Chronic Mastitis" to Carcinoma of the Breast. *Surgery Gynec. Obstet.*, **71**, 257.